# Vegetation Controls on the Spatio-Temporal Heterogeneity of Deep Moisture in the Unsaturated Zone: A Hydrogeophysical Evaluation

**DOI:** 10.1038/s41598-017-01662-y

**Published:** 2017-05-04

**Authors:** Bharat S. Acharya, Todd Halihan, Chris B. Zou, Rodney E. Will

**Affiliations:** 1 Department of Natural Resource Ecology and Management, Oklahoma State University, Stillwater, OK 74078 USA; 2 Boone Pickens School of Geology, Oklahoma State University, Stillwater, OK 74078 USA

## Abstract

Information on the spatio-temporal variability of soil moisture in the vadose zone is important to assess groundwater recharge and solute transport in unconsolidated substrate as influenced by biological processes. Time-lapse electrical resistivity imaging (ERI) was used to monitor soil moisture dynamics to a depth of 9 m in a grassland, a grassland encroached by a juniper species (eastern redcedar, *Juniperus virginiana*), a juniper woodland and an oak forest in the south-central Great Plains, Oklahoma, USA. A site-specific relationship between moisture content and electrical conductivity data was developed for the soil zone, and a perched water zone was monitored at two of the sites. Results showed that (a) change in soil moisture content was linearly correlated to change in electric conductivity in the soil zone; (b) vegetation cover type induced differences in vertical bulk electrical resistivity (ER) profiles and influenced the temporal evolution of soil moisture profiles; and (c) juniper encroachment lowered the water level in the perched groundwater aquifer. Our results suggest land use and vegetation cover type, as opposed to rock properties, controls deep water drainage for the vegetation transition zone. Methods used to measure hydrogeophysical changes, such as ERI, can be used for broader understanding of geological, physical, and biological processes and their links in Earth’s critical zones.

## Introduction

Understanding the existence and magnitude of deep drainage and water flowpaths in the vadose zone under contrasting vegetation types is critical to manage groundwater quantity and quality. Soil water content can be determined by using moisture probes or dielectric sensors, cosmic ray neutrons, gamma ray attenuation, distributed temperature sensing, and/or by using remote sensing methods^1^. However, these methods fail to provide adequate information on deep water content due to limits of coring depth^2^ and calibration techniques^1^, especially where competent rock is shallow. Electrical resistivity imaging (ERI), a non-intrusive technique, has been used since the 1830’s^3^ to characterize and monitor water distribution, contaminant plumes, contaminations and remediation, fluid transport, groundwater flow and reactions, subsurface heterogeneity and anisotropy, to map soil texture and to monitor geo-hazards^4–8^, but it’s use in monitoring vadose zone soil moisture dynamics and groundwater recharge is still limited^2, 9, 10^.

Electrical resistivity imaging is a geophysical technique which uses surface electrodes for measurement and acquisition of apparent resistivity data^7, 9, 11, 12^. Electrical resistivity (ER) data are influenced by soil particle size, form and distribution of voids, soil water content, fluid properties, and temperature^13, 14^. Temporal changes in resistivity are derived by collecting apparent resistivity from same location at different time intervals^13^. The apparent resistivity data are converted to true resistivity using an inversion model and developed into two-dimensional and three-dimensional images^7^. The temporal change in resistivity is due largely to soil water content in the absence of other subsurface reactions.

Limited studies have utilized ERI to estimate deep drainage of water and understand the dynamic interaction between vegetation and vadose zone moisture. Time-lapse ERI was used to understand the interaction between vegetation, climate and root zone moisture in a grassland-forest ecotone of Michigan, USA^15^. The grassland showed smaller change in total soil moisture content than the forest^15^. In a recent study, spatio-temporal dynamics of near-surface soil moisture was investigated using ERI in a deciduous woodland^9^. Soil moisture varied spatially, declined throughout the growing season and was negatively correlated with tree crown area and leaf area index^9^. One of the complications in using such geophysical methods is to accurately transform resistivity to soil moisture. Moisture values are underestimated when inverted resistivity values are converted into soil moisture based on petrophysical relations^16^. Despite this limitation, alternative methods for estimation of subsurface moisture distribution and migration can alter the subsurface flowpaths, so a nonintrusive method is preferred. When utilized in a comparative study between vegetation types, the relative changes in temporal ER data can be used to infer drainage relative to vegetation type instead of utilizing the tool as a quantitative soil moisture dataset.

Deep drainage and subsequent recharge of aquifers occur when soil water percolates vertically, passes the active rooting zone and enters groundwater aquifers^17, 18^. The vadose zone in water-limited regions is usually thick^19–21^ and deep percolation of water and storage in the unsaturated zone is sensitive to the depth of the rooting system^20^. Alteration in water use patterns and rooting architecture^22^ associated with changes in vegetation functional type, such as a transition between grassland and woodlands, is likely to affect the deep percolation dynamics and local recharge processes^17, 23, 24^. A global synthesis of groundwater recharge showed that grasslands produce higher recharge compared to woodlands^25^. Lower annual recharge rate was estimated after a grassland was encroached by honey mesquite (*Prosopis glandulosa*) in southwest Texas, USA^26^. While previous research indicated that encroachment by junipers (*Juniperus* spp.) reduces runoff and soil moisture in the rooting zone^27^, we currently do not know how encroachment affects deep moisture profiles which can differ due to factors such as bedrock drainage and perched aquifer in upland catchments.

**Figure 1:**
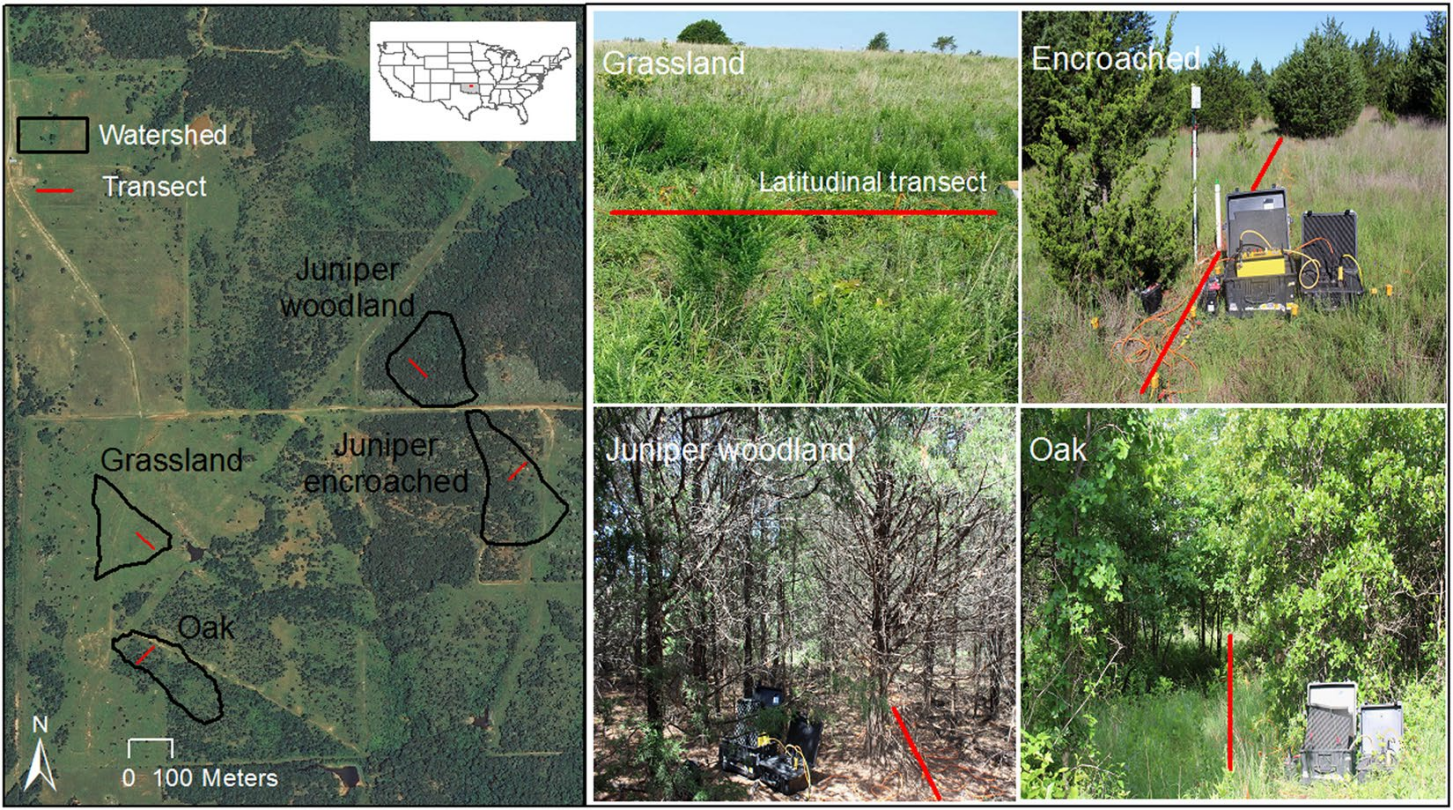
Experiment site located in the south-central Great Plains at the Oklahoma State University Cross Timber Range Research Station showing the grassland, the juniper-encroached site with SuperSting 8-channel resistivity meter and switchbox and battery, the juniper woodland and oak forest. The data used in the figure were obtained from Data Gateway (https://datagateway.nrcs.usda.gov/) and the map was created using ArcGIS 10.3.1 (Esri. Inc). The 2013 orthoimagery was photographed by USDA-FSA-APFO. Photos of different vegetation cover types were taken by Dr. Acharya and are true to color.

The objectives of this study were to (a) evaluate the ability of two-dimensional time-lapse ERI to track deep moisture changes, (b) contrast patterns of subsurface electrical resistivity to assess spatial and temporal dynamics of vadose zone moisture under four different vegetation types (grassland, juniper-encroached prairie, juniper woodland, and oak (*Quercus spp*.) forest; Fig. 1 and (c) evaluate how juniper encroachment affects water level in the perched groundwater aquifer in the south-central Great Plains, USA. Results will be used to help assess the effects of vegetation types on deep recharge potential. This information will allow us to better estimate changes from land use and land cover on a previously poorly understood portion of the water cycle and discern geological, physical, and biological processes.

## Results

### Relationship between electrical conductivity and soil moisture

**Figure 2:**
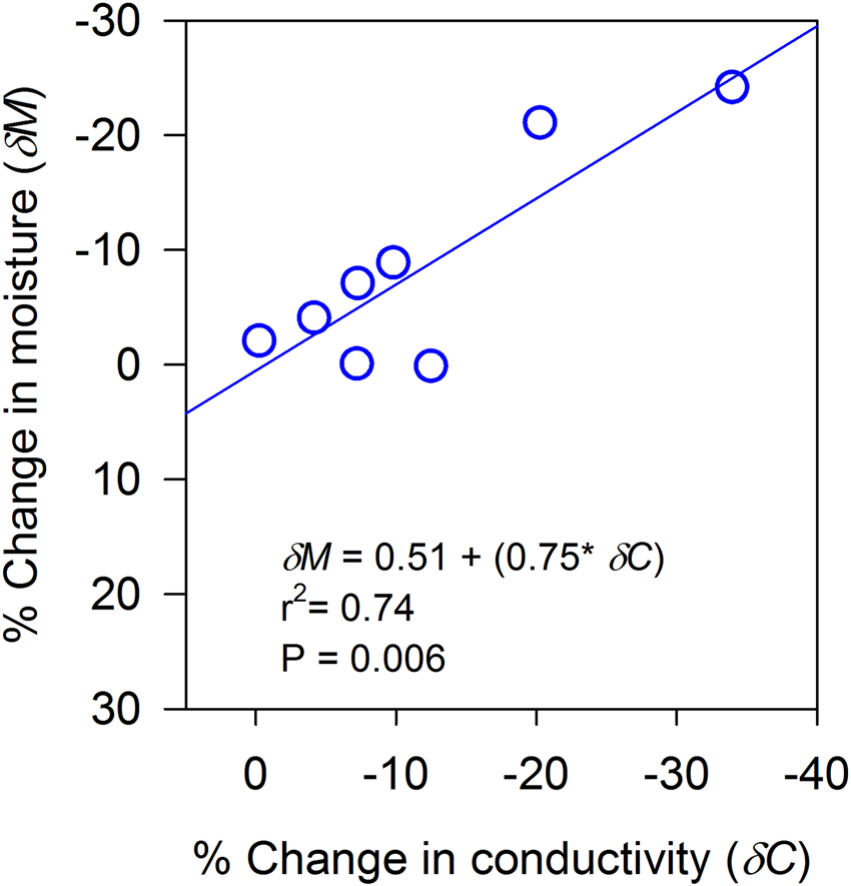
Direct linear relation between change in moisture (0–12 cm) and change in conductivity (10–25 cm) after inversion with data from just below the soil surface in the inverted datasets in soil profile across all vegetation cover types in the experimental site.

Percent change in soil moisture content was linearly related to change in bulk electrical conductivity in the top 25 cm (p = 0.006, r^2^ = 0.74) of a soil profile. The standard error of estimate was 5.19% (Fig. 2).

### Electrical properties under four vegetated areas

#### Background resistivity images

**Figure 3:**
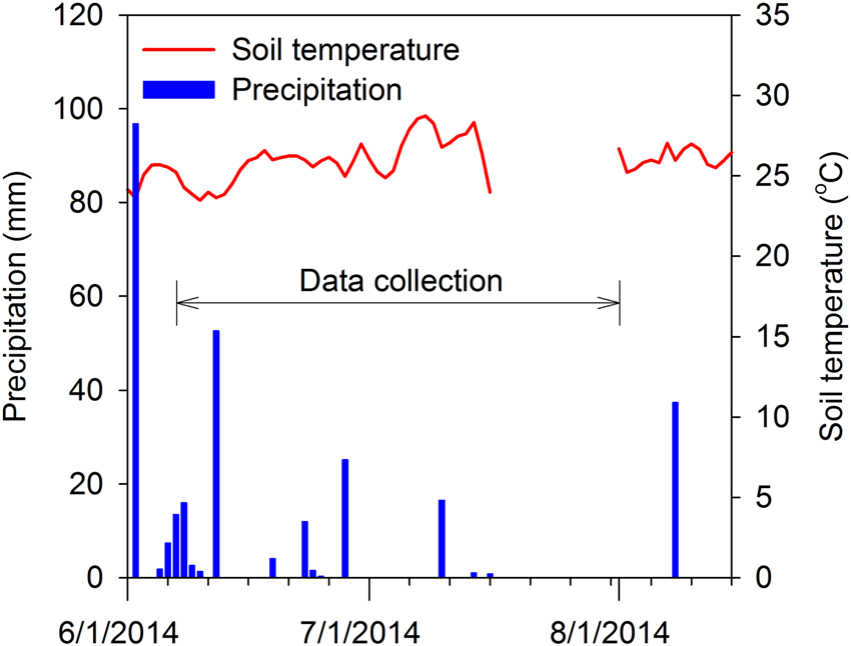
Daily precipitation in mm (TB3 siphoning tipping bucket rain gauge with a 0.254 mm tip; Hydrological Services America, Lake Worth, FL) and soil temperature in °C (107-L temperature probe; Campbell Scientific, Logan UT) from a weather station at Cross-Timber Range Research Station during June to August 2014. Soil temperature values at 5 cm depth are averaged over 5 minute and recorded. Horizontal arrow indicates the time of ERI data acquisition.

**Figure 4:**
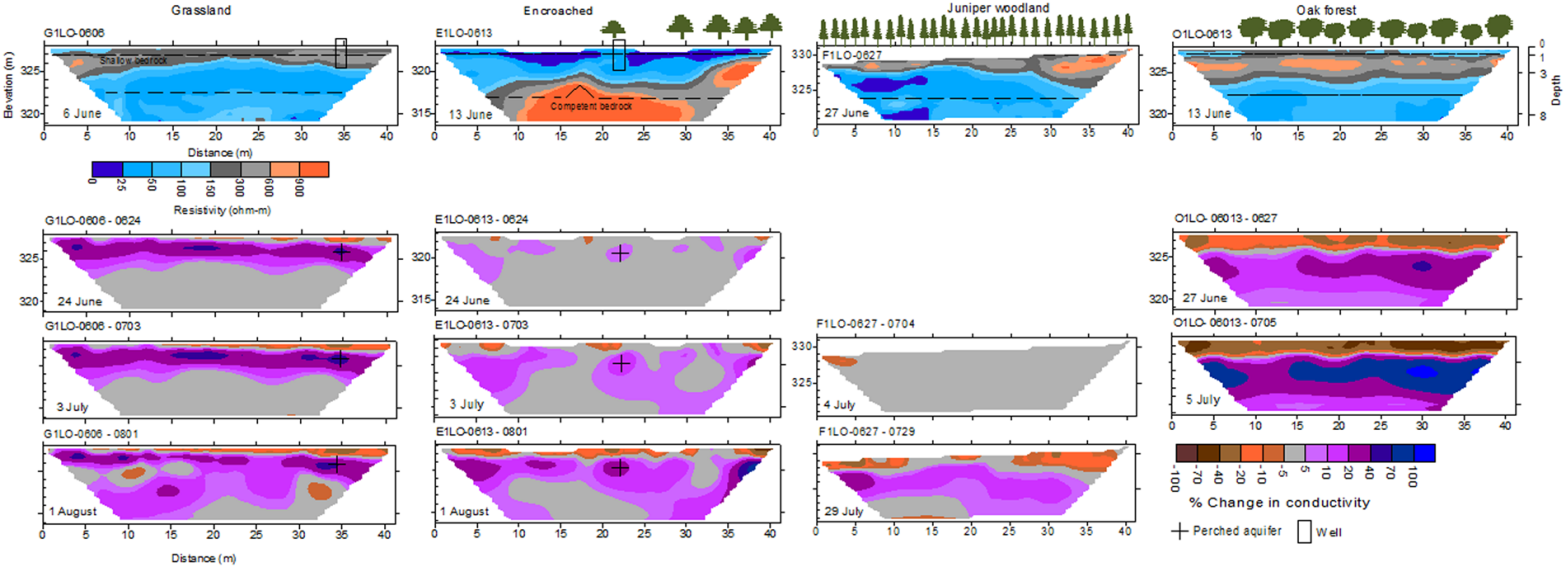
Electrical resistivity images for a grassland, a grassland encroached by a juniper species (eastern redcedar, *Juniperus virginiana*), a juniper woodland and an oak forest. Top images are background images of resistivity and subsequent images are transient images shown as % change in conductivity. A positive change in conductivity corresponds to higher conductivity and higher moisture. Images were developed from latitudinal transects deployed with 56 electrodes between June and August 2014.

The grassland site received 97 mm of rain four days prior to ER data acquisition followed by periods of no-rain indicating a wetting-drying cycle (Fig. 3). Background ERI from the grassland site showed a resistive layer (100 to 600 Ω-m) running across the line to a depth of 3.5 m (Fig. 4). The critical zone below 3.5 m depth had lower resistivity (25–100 Ω-m) along the 42 m long profile. The juniper-encroached site showed lower resistivity (0 to 100 Ω-m) to a depth of 3.5 m. Resistivity values below 6 m were higher ranging from 300 to 900 Ω-m (Fig. 4). The background image from the juniper woodland exhibited greater resistivity values to a depth of 2.5 m (150 to 900 Ω-m) followed by lower resistivity values to the depth of 9 m (0 to 100 Ω-m). The background electrical properties in oak forest showed higher resistivity up to 4.5 m depth (100 to 600 Ω-m) and lower resistivity below 4.5 m (25 to 100 Ω-m); a resistivity distribution pattern similar to the grassland site.

#### Transient conductivity images

Transient conductivity images showed a layered moisture migration profile, non-wetted and wetted irrespective of vegetation types, because there was positive change in conductivity below 3 m soil depth which indicated increased moisture at deeper depths (Fig. 4). Vegetation induced differences in vertical bulk ER profiles. The percent change in conductivity for the grassland and oak forest sites was negative in the top soil layer but positive below 3 m depth indicating soil drying near the surface. For the grassland site, the top soil mantle in the transient images between June (G1LO-0606-0624) and August 2014 (G1LO-0606-0801) exhibited up to a 40% decrease in conductivity at the lateral distance of 14 to 42 m from the left hand side (LHS) (Fig. 4). However, conductivity increased by 5 to 40% below 1 m depth. The juniper-encroached site showed negative change in conductivity (decrease in soil moisture) near the soil surface in June 2014 (E1LO-0613-0624) and the anomaly further enlarged in subsequent pseudosections of July (E1LO-0613-0703) and August 2014 (E1LO-0613-0801) (Fig. 4). A meter thick near-surface soil layer showed up to 70% decrease in conductivity. Two circular nuclei near 321 m elevation from LHS of the pseudosection E1LO-0613-0801 showed an increase in conductivity (increased soil moisture) by 20%. For juniper woodland, the first transient image from 04 July 2014 showed no change in conductivity except a small patch at the left corner of image, which clearly exhibits 10 to 20% decrease in conductivity. At the end of July, conductivity decreased (decreased soil moisture) by 5 to 70% in the top 3 m and increased (increased soil moisture) by 5 to 20% below 3 m soil depth.

For the oak site, there was downward propagation of high soil moisture zone because conductivity increased over time at depths below 3 m. The first two transient images from 27 June and 05 July 2014 in oak forest site showed 20 to 100% decrease in conductivity up to 2.5 m depth, and 0.5 m thick gray region delimited the zone with no change in conductivity. However, the conductivity increased by 5 to 70% between 3 and 8 m depth of the pseudosection O1LO-0613-0705 in July 2014.

### Temporal variability in water level

**Figure 5:**
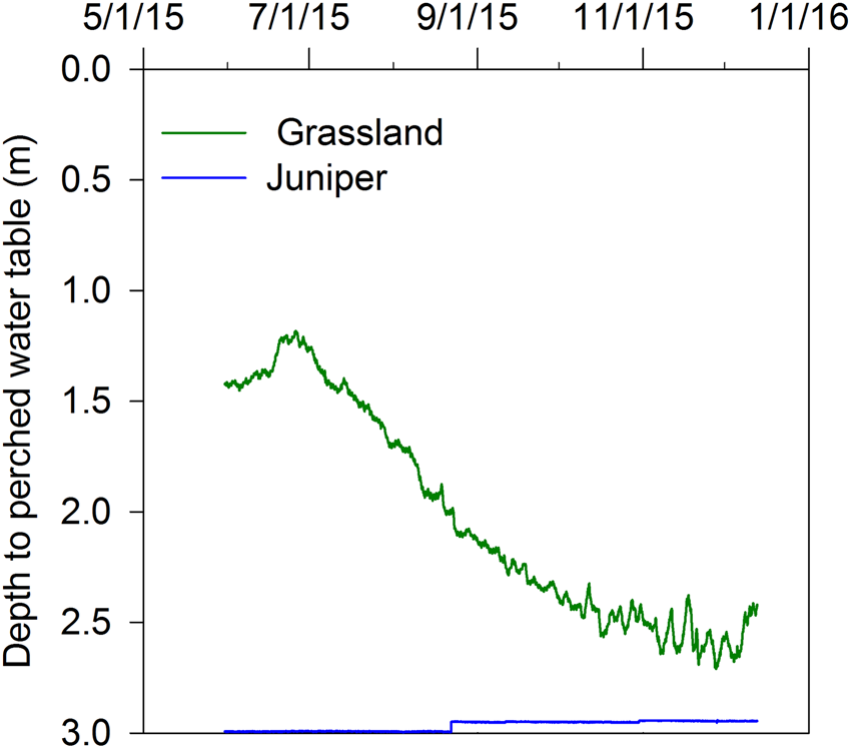
Water level recorded at 15-minutes interval during 31 May to 12 Dec 2015 from two monitoring wells of 3 m depth in a grassland and a juniper-encroached site. Wells were drilled in hydraulically conductive location inferred from ERI and piezometers were instrumented with CTD-10 sensors.

The level of the perched water table fluctuated between 1.2 to 2.6 m under the grassland site which was higher than the water level under the juniper-encroached site which fluctuated between 2.7 and 3.0 m during our study period. Peak water level was recorded on 25 June 2015 in the grassland, but the water level in the juniper-encroached site peaked during 28 November 2015 (Fig. 5). The perched aquifers showed little to no change in conductivity (Fig. 4).

## Discussion

Spatio-temporal dynamics of soil moisture content affects evapotranspiration, deep drainage and local recharge processes. Electrical resistivity is inversely related to soil moisture content and this relationship is largely controlled by soil physico-chemical properties such as texture, particle size and geometry of pores (void distribution and form), and pore fillings^14, 22, 28^. The relationship is therefore soil-specific and follows linear or non-linear relationships that can include second order polynomial, power, exponential and logarithmic relationships^28^.

We established a field-based relationship between bulk electrical conductivity and moisture content in the top 25 cm of a soil profile (Fig. 2) as saturation soil water conductivity in laboratory data will differ from field-derived bulk electrical conductivity^16^. Changes in bulk electrical conductivity provide a semi-quantitative measure of vadose zone moisture. However, this field relationship will change for deeper layers due to differences in soil compaction, lithification, anisotropy due to layering and differences in clay and organic matter^29^. We assume that the relationship is valid for the soil zone, but that for the bedrock zone, we only assume that a positive change in bulk conductivity indicates the addition of water to the bedrock in that area.

We collected ER data on the juniper-encroached site and the oak forest on the same day (13 June 2014) and both sites received 53 mm of rainfall a day prior to the ER data acquisition (Fig. 3). However, background resistivity in the oak forest indicated a higher potential than juniper-encroached site for deep drainage of infiltrated water. Studies indicate that trees play an important role in increasing the preferential flow of water^29^, and improving soil infiltration capacity^30^. Crown area and leaf area index in deciduous forests such as oak are negatively correlated to soil moisture at the beginning of growing season, but water content may increase with no transpiration and higher throughfall after leaves abscise^9^. However, the accrual of plant litter in juniper-encroached catchments and oak forest is also associated with interception loss of rainfall, hydrophobicity (e.g. juniper litter), lower surface moisture content, and unsaturated hydraulic conductivity^31, 32^. Juniper trees have high interception ratio, greater plant water uptake, potential to access deep moisture, and therefore alter soil hydrologic properties and reduce downward flux of water^33^.

Time-lapse images are necessary to understand the influence of weather and vegetation on rapidly changing variables such as subsurface moisture distribution^15^. A decreased conductivity was observed in the upper 0.5 to 1.0 m of soil mantle in the July 2014 ER image (G1LO-0606-0703) which received a total of 136 mm rainfall between 6 June and 3 July 2014, implying that most of the water was either used by plants, lost to evaporation or drained deeper within the profile. A drier layer was observed near the surface following a rainfall event four days prior to ER measurement in a grassland in Michigan, USA, wherein water drained deeper into the profile because soil nearly attained field capacity^15^. Our results also indicate patterns of potential lateral flow in the grassland cover type. We were limited by the transect number to confirm lateral flow but barriers to vertical flow of water by argillic horizons and ponding of water over the confining layers with low permeability may result in perched water tables, and contribute to the development of lateral flow^34, 35^.

Most of the juniper trees in the juniper-encroached site were towards the end of pseudosection. A substantial part (>50%) of this latitudinal transect was free of vegetation, and thus limited canopy cover and juniper litter was present along the distance up to 35 m to intercept rainfall^32^ (Fig. 4). Regions devoid of trees performed similar to the grassland site with reduced conductivity (lower soil moisture) on the near-surface but increased conductivity (higher soil moisture) at deeper layers. Areas directly and/or closely below the trees had reduced conductivity (lower soil moisture) as observed near the center and towards the end of pseudosection in July (E1LO-0613-0703) and August 2014 (E1LO-0613-0801). Trees can increase infiltration^30^, redirect and funnel intercepted water into the soil as stemflow^36^ and facilitate downward movement of water^29^, thus increasing conductivity at the deep unsaturated zone. Similarly, we observed small storm events ranging from 0.2 to 17 mm prior to ER data acquisition in July and August. These small storm events in partly moist soils (Fig. 2) can significantly alter deep drainage by increasing hydraulic and physical connection between pores in soil. Plant water use by individual juniper trees, as indicated by sap flow measurement, ranged from 2 to 80 liters per day, which was largely explained by tree characteristics, environmental variables (temperature and solar radiation), and volumetric moisture in the upper 10 cm of the soil^37^.

Transient images in the oak forest indicate deep drainage of infiltrated water (Fig. 4). Higher electrical conductivity and a stable moisture profile were observed in tree locations in an oak-pine forest in New Jersey after a rain event, suggesting the cumulative role of roots and water potential gradients on soil moisture regulation^38^. Our oak forest site was not previously cultivated but catchments encroached by juniper were cultivated prior 1950s’ and soils may have been compacted. Soil bulk density in the top 30 cm in the juniper catchments was estimated to be 1.25 g cm^−3^ which is relatively higher than the grassland catchment (1.19 g cm^−3^)^27^. The surface was dry in the juniper woodland (Fig. 4), which may result from canopy and/or litter interception of rainfall^32, 39^, but after a large rain event, water is likely to infiltrate deep as indicated by conductive subsurface below 3 m. It is important to mention that one transect was used for each cover type in this study. It is therefore likely that some of the differences are due to the specific soil types and conditions that were under the specific transect, not the vegetation. However, the length of transect enabled us to capture spatial heterogeneities in soil types, and thus were representative of the soil types.

Water level in two monitoring wells (Fig. 5) indicated that vegetation can modulate recharge processes, and woody plants can decrease the water table in a perched aquifer. A sandstone contact below 1 m depth allowed the development of a perched water table in the bedrock. The regional water table is located at approximately 12 m below land surface. The results are akin to and support electrical conductivity from the time-lapse ERI. Woody plants intercept rainfall via plant canopy and litter^32, 36^, affect ET differently^40^ and/or tap into deep water to reduce water level^41, 42^. Water extraction can, however, vary with vegetation structure, slope positions and depth of water table. For example, cerrado woody plant community in a neotropical savanna in Brazil uses a minimum of 30% of total water from deeper layers^42^. Woody plants may rely on shallow soil water during the wet season and deep water during the dry season^42, 43^.

Woody plant encroachment in grasslands and savannas is a global phenomenon, which results in biogeochemical, ecophysiological and ecohydrological changes^44–48^. Grasslands in the south-central Great Plains are rapidly being transformed from herbaceous-dominated systems to woodlands or woody-dominated savanna^49, 50^. Proliferation of woody plants into rangelands previously dominated by herbaceous plants in Oklahoma, USA^51^ is associated with increased ET due to greater leaf area, deeper roots, and greater canopy interception, and potential loss of water available for streamflow and recharge, especially in the semi-arid and sub-humid regions^52–54^. Changes in electrical conductivity, and spatio-temporal variability in groundwater level are therefore important to understand the effects of land-use and vegetation cover on deep water dynamics. Our study shows that the ERI technique can differentiate soil moisture as related to vegetation differences over large transects reaching throughout the vadose zone and the transects can be repeatedly measured to determine change over time without altering the native soil or hydrological flow patterns.

## Conclusion

The results contextualize and highlight the ability of time-lapse ERI to detect deep moisture dynamics and localized recharge sites. Vegetation affects the temporal evolution of soil moisture profile in the vadose zone. Vegetation transition such as juniper encroachment into a grassland is likely to reduce the potential of deep recharge. Understanding the impacts of vegetation and vegetation transition is a key to manage land cover for streamflow and groundwater recharge in water-limited ecosystems. Future studies should incorporate soil hydraulic properties to improve the capability of ERI to analyze water distribution and solute transport as influenced by biological processes along with the use of hydrological models such as HYDRUS 2D/3D.

## Methods

### Experimental site

The study was conducted at the Cross-Timber Range Research Station, which is owned and managed by Oklahoma State University. The study area is located about 11 km southwest of Stillwater, Payne County, Oklahoma, USA (36.06°N, 97.18°W, and 331 masl) in the lower Cimarron River watershed. Based on long-term climate data (from 1971 to 2000), the site has a sub-humid climate with mean annual temperature of 15.5 °C, and mean annual precipitation of 948 mm^32^.

### Soil and bedrock structure

Across the four sites, the soil and bedrock structure are similar. Two major contacts were observed on the sites, a shallow weathered bedrock at 1 m and a competent bedrock at 6 m depth. The soil was tested across the four sites on numerous occasions and is approximately 1 m deep. At this depth, the visual contrast with the underlying bedrock is not significant, but hand augers will not penetrate below this depth. The bedrock cannot be penetrated with direct push tooling either, but can be evaluated using a direct push auger rig (Geoprobe 6200 TMP, Kejr, Inc. Salina, KS). The auger can advance through the weathered rock body to approximately 6 m at which point the rock is too competent for augers. Dominated by the Wellington formation of Permian age, the geology of the experimental site largely consists of red-brown shale, fine-grained sandstone and mudstone conglomerate (http://www.owrb.ok.gov/). Major soil types in the study site include Stephenville-Darnell complex, Grainola-Lucien complex, and Coyle soil series approximately 1 m thick. Stephenville are fine-loamy, siliceous, active, thermic Ultic Haplustalfs; Darnell are loamy, siliceous, thermic, shallow, Udic Ustochrepts; Grainola are fine, mixed, thermic, Vertic Haplustalfs; Lucien are shallow fine sandy loam, mixed, thermic, shallow Typic Haplustolls; and Coyle series are fine-loamy, siliceous, thermic, Udic Argiustolls^55, 56^.

### Vegetation types

Two major vegetation types in the site include grassland (tallgrass prairie) and oak woodland. Grassland areas consist of C_4_ grasses, including little bluestem (*Schizachyrium scoparium*), big bluestem (*Andropogon gerardii*), Indiangrass (*Sorghastrum nutans*), switchgrass (*Panicum virgatum*), and tall dropseed (*Sporobolus asper*)^57^. Major forbs include western ragweed (*Ambrosia psilostachya*) and broomweed (*Gutierrezia dracunculoides*). Grasses are often found on Grainola-Lucien complex and Coyle soil series. Oak woodland is associated with Stephenville-Darnell complex and is dominated by post oak (*Quercus stellata* Wangenh.), blackjack oak (*Q*. *marilandica* Muenchh.)^58^. In recent years, a juniper species (*J*. *virginiana*, eastern redcedar) is rapidly encroaching and expanding in tall- and mixed-grass grasslands, and converting grassland patches into juniper woodlands. In some locations, the canopy coverage by eastern redcedar reaches nearly 100% with live branches beginning several meters aboveground, while in some other locations, juniper trees are widely spaced with the live branches distributing along the entire stem^37^. Four experimental catchments, grassland, juniper-encroached, juniper woodland and oak forest were selected for the study. The catchments are between 2.0 and 2.7 ha with a mean slope between 5 and 6% (Fig. 1).

### Electrical Resistivity Imaging (ERI)

Electrical resistivity in the subsurface can be quantified using a multielectrode array to collect apparent resistivity data, which can then be inverted into model or true resistivity values of the subsurface (Advanced Geoscience, Inc. SuperSting 8-channel resistivity instrument). Low-frequency alternating current is induced in two current electrodes and the potential difference is measured between two electrodes in an induced electric field. Typical range of current varies from 100 to 300 mA in these experiments. Contact resistance tests were completed prior to initiating each survey to identify poor electrical contact between the electrodes and soil. We observed strong electrical contact between the electrodes and soil. Apparent resistivity ${\rho }_{a}$ (Ω-m) is defined as the ratio between measured potential difference (*∆V*) and induced electric current (*I*) into the ground.

1 $${\rho }_{a}=\frac{{\rm{\Delta }}V}{I}$$

Soil bulk electrical conductivity $\sigma$ (Sm^−1^) is defined as the reciprocal of resistivity:

2 $$\sigma =\frac{1}{\rho }$$

Subsurface bulk electrical conductivity is significantly influenced by different factors such as grain size, porosity, degree of water saturation, and concentration of dissolved salts^22, 24^.

The permanent latitudinal transects were 42 m long and oriented along the surface topography contour lines. All of the transects were located and deployed with electrodes of 48.3 cm length and 1.6 cm diameter made up of copper coated steel lightning rods in June 2014. Electrode installation was completed a week prior to first ERI measurement to ensure good contact between soil and electrodes. Installation of permanent electrode lines prevents and/or minimizes any alterations in near-surface soil properties^59^. Thus, a total of 56 electrodes were permanently deployed on the surface across each latitudinal transect with 0.75 m electrode spacing.

### Surface soil temperature

Diurnal and seasonal fluctuations in soil temperature can alter bulk electrical resistivity data (ER data)^60^ by decreasing pore fluid resistivity and increasing the mobility of ions^2, 14^. Surface soil temperature was measured randomly across the transects in proximity to electrodes to a depth of 12 cm using a reference thermometer (Thermoworks, USA) (*accuracy* + 0.05 °C) to detect temperature variability, and to determine the necessity of temperature correction for resistivity values. Daily soil temperature averaged over 5 minutes was also reported from a nearby weather station. We did not correct resistivity values for temperature because as diurnal soil surface temperature minimally fluctuated (max = 28 °C, min = 24 °C, mean = 26° ± 1.3 °C SD) throughout the months of data acquisition (Fig. 3).

### Transect locations and description

A digital elevation model (DEM) was generated for the site from Light Detection and Ranging (LiDAR) bare earth elevation dataset-2 m for projected in North American horizontal datum of 1983 obtained from USDA NRCS. DEM’s produced from LiDAR dataset are of higher resolution and provide greater accuracy for base layer for terrain mapping, watershed evaluations and hydrological modelling. The vertical accuracy of the LiDAR bare earth elevation dataset, expressed as the root mean square error, was approximately 12.5 cm (G. Utley, personal communication, NRCS, OK, January 13, 2014). Transects were selected to parallel the topography along contours to ensure that the soil profile was perpendicular to the flow paths and to adequately represent the vegetation cover. In this study, the transect for juniper and oak woodland represented trees growing in a closed-canopy stand and the woody canopy coverage in juniper woodland was nearly 100% and in oak forest was >80% with branches that occurred on the upper trunks only^32, 36, 37^ (Fig. 1). The transect for encroached site represented an estimated woody canopy coverage of approximately 40–50% with different size juniper trees (height = 1.5 to 5.5 m and diameter measured 1.37 m the soil surface = 0.2 to 14.9 cm; Fig. 1).

### Acquisition of apparent resistivity

Apparent resistivity data were collected following rainfall events in June 2014 and after drier conditions in June, July and August 2014 to understand and image deep drainage of water. The 42 m long ERI line provided data acquisition to infer subsurface processes and anomalies and deep moisture to the depth of approximately 9 m. Hundreds of data points were collected in an automated mode following Oklahoma State University (OSU) proprietary method (the Halihan-Fenstemaker, HF, method, OSU, 2004). The HF method provides increased sensitivity of subsurface images than the standard dipole-dipole array by approximately an order of magnitude^10, 61^. The resolutions of electrical resistivity images were half of the stake spacing (0.375 m). Data collection included repetitive measurements. The average error in apparent resistivity data was 0.4%.

### Bulk Conductivity and soil water content

Surface soil moisture (0–12 cm) near electrodes was determined using HydroSense II (Campbell Scientific, USA) during ERI data acquisition. We established a relationship between change in conductivity after inversion with data from just below the surface in the inverted datasets (10–25 cm) and change in soil moisture (0–12 cm) in the soil profile (Fig. 2) on the basis of *in situ* monitored data. Electrical resistivity was inversely related to electrical conductivity (see equation 2). Soil moisture data from the same location were compared at different dates to determine changes in soil moisture content. The relationship between change in soil moisture and conductivity was primarily based on shallow soil depth and therefore may change for deeper layers. The assumption utilized in this work is that the relationship between the soil and bedrock may vary, but the changes in bulk conductivity indicate changes in soil moisture as measured near the surface. Thus, the resulting changes in conductivity provide a qualitative measure of bedrock vadose zone soil moisture content changes.

### Temporal variability in water level

Two monitoring wells of 3 m depth and 5.1 cm diameter were drilled in two areas with lower electrical resistivity interpreted as a hydraulically conductive location. One well was drilled in the grassland and another in the juniper-encroached site using a trailer-mounted Geoprobe (6200 TMP, Kejr, Inc. Salina, KS). The wells were instrumented with CTD-10 sensors which were connected to an EM50 data logger (Decagon, Pullman, WA, USA) to measure water level (*accuracy* + 0.05%) at 15-minutes intervals.

### Data analysis

Field data were corrected for topography to adjust the change in distance between the electrodes^62^. A TOPCON Hyperlite Plus Global Positioning System (TOPCON Positioning System Inc., Livermore, CA, USA) was configured with a base station and a GPS rover with Bluetooth connected handheld unit, and latitude, longitude and elevation for each electrode were recorded with 1 cm accuracy. The location of each electrode was thus corrected based on the easting, northing and elevation of base station obtained from Online Positioning User Service (overall root mean square error <3 cm, peak to peak errors <5 cm), which is operated by National Oceanic and Atmospheric Administration (https://www.ngs.noaa.gov/OPUS/).

The relationship between change in soil moisture content and electrical conductivity in soil profile was analyzed using regression analysis at a significance level of α = 0.05. Data quality was evaluated using *r* ^*2*^ and root mean square error (RMSE). Pseudo-sections of electrical resistivity images were developed in two-dimensions using an inversion and differencing protocols described in other work^6, 7^. Random noise error was eliminated prior to inversion iterations to prevent extreme values. Data repeatability error in excess of 2% was eliminated by removing values prior to inversion. The apparent resistivity data collected in field were inverted to create a model space of resistivity values to replicate the collected data^7^. The later values are also called true resistivity values. The root mean square (RMS) inversion error was reported in percent for pseudo-sections of electrical resistivity to illustrate goodness of fit. The lower RMS inversion error represents better input data, inverted model and model fit. The RMS inversion error ranged from 3.82 to 5.07% for the ER data. Given that the lithology and soil texture were unchanged, background ERI was used to compare and characterize spatio-temporal variability in subsurface resistivity. Background ERI provided initial images of resistivity based on inversion model from measurements taken at time step 1. They refer to reference pseudo-sections developed prior to actual measurements to better describe temporal anomalies in resistivity^6, 63^. Temporal data were generated by differencing subsequent ER datasets and determining changes in conductivity with positive changes corresponding to higher conductivity and higher moisture. Images were contoured using Surfer (Golden Software Inc), and presented with a consistent color scheme.
